# Lifestyle and Dietary Habits Affect Plasma Levels of Specific Cytokines in Healthy Subjects

**DOI:** 10.3389/fnut.2022.913176

**Published:** 2022-06-24

**Authors:** Vittoria D'Esposito, Michele Francesco Di Tolla, Manuela Lecce, Francesco Cavalli, Michele Libutti, Saverio Misso, Serena Cabaro, Maria Rosaria Ambrosio, Alessia Parascandolo, Bianca Covelli, Giuseppe Perruolo, Mario Sansone, Pietro Formisano

**Affiliations:** ^1^URT “Genomic of Diabetes”, Institute of Experimental Endocrinology and Oncology (IEOS), National Research Council (CNR), Naples, Italy; ^2^Department of Translational Medical Sciences, University of Naples “Federico II”, Naples, Italy; ^3^The Anti-inflammaging Company AG, Zurich, Switzerland; ^4^Oncology Department, Azienda Sanitaria Locale Napoli 3 Sud, Naples, Italy; ^5^Unit of Transfusion Medicine, Azienda Sanitaria Locale Caserta, Caserta, Italy; ^6^Department of Electrical Engineering and Information Technology, Polytechnic and Basic Sciences School, University of Naples Federico II, Naples, Italy

**Keywords:** cytokines, low-grade chronic inflammation, biomarkers, body mass index, inflammaging

## Abstract

Low-grade chronic inflammation (LGCI) is a common feature of non-communicable diseases. Cytokines play a crucial role in LGCI. This study aimed to assess how LGCI risk factors [e.g., age, body mass index (BMI), smoke, physical activity, and diet] may impact on specific cytokine levels in a healthy population. In total, 150 healthy volunteers were recruited and subjected to questionnaires about the last 7-day lifestyle, including smoking habit, physical activity, and food frequency. A panel of circulating cytokines, chemokines, and growth factors was analyzed by multiplex ELISA. BMI showed the heaviest impact on the correlation between LGCI-related risk factors and cytokines and was significantly associated with CRP levels. Aging was characterized by an increase in IL-1b, eotaxin, MCP-1, and MIP-1α. Smoking was related to higher levels of IL-1b and CCL5/RANTES, while physical activity was related to MIP-1α. Within the different eating habits, CRP levels were modulated by eggs, red meat, shelled fruits, and greens consumption; however, these associations were not confirmed in a multivariate model after adjusting for BMI. Nevertheless, red meat consumption was associated with an inflammatory pattern, characterized by an increase in IL-6 and IL-8. IL-8 levels were also increased with the frequent intake of sweets, while a higher intake of shelled fruits correlated with lower levels of IL-6. Moreover, IL-6 and IL-8 formed a cluster that also included IL-1b and TNF-α. In conclusion, age, BMI, smoke, physical activity, and dietary habits are associated with specific cytokines that may represent potential markers for LGCI.

## Introduction

Low-grade chronic inflammation (LGCI) is a common characteristic of many non-communicable diseases, such as obesity, type 2 diabetes, cardiovascular disease, chronic respiratory disease, and cancer. These frequent disorders are chronic conditions and are responsible for ~71% of all global deaths (1, 2).

The LGCI has been recognized as a distinct type of inflammation. It is not accompanied by inflammatory classical signs and is not usually driven by pathological stimuli. LGCI is triggered by sentinel cells (i.e., macrophages and dendritic cells) that monitor for tissue stress and malfunction and, together with cells and molecules of the innate immune response, orchestrate the restoration of the normal/optimal homeostatic state (3). However, LGCI often does not resolve in a timely and controlled way and becomes chronic and smoldering (4).

Multiple cytokines preside over LGCI evolution. The cytokine network is a highly complex system that includes not only interleukins, chemokines, interferons, and tumor necrosis factors but also cellular and soluble receptors and serum mediators. Cytokines are small, non-structural proteins with pleiotropic effects. They have pro-inflammatory and/or anti-inflammatory functions, and some of them are able to control their own production (4–6).

Obesity, aging, tobacco use, physical inactivity, and unhealthy diets are considered the main risk factors for LGCI. These physiologic, environmental, and/or behavioral stimuli modify the cellular homeostasis, leading to cell stress and to the production of cytokines (3, 4, 7).

The association between obesity and LGCI is the most largely documented. Obesity drives the pathological expansion of the adipose tissue, which harbors enlarged hypertrophic adipocytes, impaired vasculogenesis, and enhanced fibrosis and hypoxia. Dysfunctional adipose tissue secretes pro-inflammatory cytokines that promote local and systemic inflammation, contributing to the onset of obesity-related diseases (8–10). The loosening of the cytokine balance between the pro-inflammatory and anti-inflammatory control is a characteristic feature also of aging. It has been defined as “inflamm-aging” and takes part in all aging-related diseases (4). In addition, smoking was found independently involved in the progression of LGCI, while physical activity has been recognized as an instrument to modulate LGCI (11, 12), although intense physical exercise is often paralleled by enhancement of inflammatory factors (13). Finally, it is now clear that dietary intake regulates inflammation through the complex interactions between foods and nutrients with bioactive properties. In this regard, several dietary indexes have attempted to assign inflammatory scores to specific foods/nutrients (14–16). Some nutrients have been classified as anti-inflammatory, and other nutrients have been defined as pro-inflammatory, according to their ability to promote the release of specific mediators (1, 7, 15).

Thus, cytokine levels may vary upon a plethora of stimuli. To date, critical levels of cytokines as biomarkers have not been defined, both in classical inflammatory diseases and in LGCI. Furthermore, only few studies have been performed to investigate cytokine levels in healthy subjects, and a limited number of LGCI-related risk factors have been explored when considering healthy subjects' cytokine profiles (5, 6, 17).

In this study, we have analyzed the cytokine profile in a cohort of healthy volunteers. We have assessed how gender, age, BMI, smoking, physical activity, and diet may modify specific cytokine levels potentially contributing to LGCI.

## Materials and Methods

### Population Enrollment and Serum Collection

In total, 150 blood donors were recruited at the Transfusion Medicine Unit, Azienda Sanitaria Locale, Caserta, Italy, from January to July 2019. Exclusion criterion was ineligibility to donate blood, as indicated in D.M. 2/11/2015 (i.e., documented infectious diseases or other proliferative, degenerative, and autoimmune diseases; altered blood count and blood pressure; and drug assumption). The sample size was representative of the population of eligible donors, calculated on annual base. Since this is a pilot study with an exploratory nature, the power analysis has not been performed. All volunteers enrolled underwent detailed clinical phenotyping, including measurement of height, weight, and waist. Body mass index (BMI) was calculated as ratio of body weight (kg)/height (m^2^). Moreover, a detailed questionnaire about anamnestic and anthropometric data, smoking habit, physical activity, and weekly food frequency was administrated to each subject (as described below). A serum sample from all donors was obtained and stored at −20°C. Transfusion Medicine Unit performed cytometric blood counts and biochemical analyses (AST, ALT, cholesterol, triglycerides, iron, total proteins, creatinine). Investigations were carried out following the rules of the Declaration of Helsinki of 1975, revised in 2013. Informed consent was obtained from every volunteer before the procedure. The protocol was approved by the ethical committee of the University of Naples (protocol no. 349/18).

### Lifestyle Questionnaire

Lifestyle questionnaire was face-to-face administered by an expert nutritionist and included information about the last 7 days of physical activity and food frequency. The physical activity of enrolled volunteers was recorded and analyzed with the short form of the International Physical Activity Questionnaire (IPAQ) (18). The questionnaire reports the activity of four intensity levels: (1) vigorous-intensity activity, such as aerobics; (2) moderate-intensity activity, such as leisure cycling; (3) walking; and (4) sitting. For each activity, the *Metabolic EquivalenT (MET)*, a unit used to express energy spent and oxygen burned, was automatically calculated. MET value is given by the following equation (19):


$$\begin{array}{l} MET=\frac{3.5\;ml\;\left(O_{2}\right)}{bodyweight\;\left[Kg\right]\;\times \;time\;\left[h\right]\;} \end{array}$$

Based on the MET data analysis, as suggested by Craig et al. (18) and by the Guidelines for Data Processing and Analysis of the IPAQ (www.ipaq.ki.se), and considering MET value distribution in the enrolled population, volunteers have been classified into three groups, namely, volunteers with low or null physical activity (MET <1,000), volunteers with an average physical activity (1,000 < MET ≤ 3,000), and volunteers with an intense physical activity (MET > 3,000).

Dietary information was collected with a weekly food-frequency questionnaire (FFQ) conceived on that used in the framework of the Italian EPIC study (20, 21). Accordingly, a list of foods was developed in line with the local food availability, culturally specific and dietary habits (Mediterranean diet). Food frequency type with portion size was estimated by means of pictures. The participants were asked to report the frequency of consumption of food items listed in Supplementary Table S1. Pictures showing different portion sizes – arranged by increasing amount – followed the question on frequency and corresponded to a specific portion in grams. Additional questions addressed issues such as habitual cooking practices and types of cooking fats. Questionnaires were finally digitalized, and data relative to the weekly total consumption of each food item, expressed in grams, were gathered and analyzed.

### Determination of Cytokines, Chemokines, and Growth Factors

Serum samples were screened for the concentration of interleukin (IL)-1ra, IL-1b, IL-2, IL-4, IL-5, IL-6, IL-7, IL-8, IL-9, IL-10, IL-12 (p70), IL-13, IL-15, IL-17A, basic fibroblast growth factor (FGF), eotaxin, granulocyte-colony stimulating factor (G-CSF), granulocyte-macrophage colony-stimulating factor (GM-CSF), interferon-γ (IFN-γ), interferon-γ inducible protein 10 (IP-10), monocyte chemoattractant protein-1 (MCP-1), macrophage inflammatory protein-1 (MIP-1) α, MIP-1β, C-C motif chemokine ligand 5 (CCL5)/RANTES, TNF-α, platelet-derived growth factor (PDGF-BB), and vascular endothelial growth factor (VEGF) using the Bio-Plex Multiplex Human Cytokine, Chemokine, and Growth Factor Kit (cat. n. M500KCAF0Y, Bio-Rad, Hercules, CA, USA) according to the manufacturer's protocol, as previously described (17, 22). The magnetic bead-based assay was performed on a Bio-Plex 200 System (Bio-Rad, Hercules, CA, USA). All the values obtained were included within the detection limits indicated by the manufacturer (bio-rad.com/Bio-Plex/AnalyteGuide). High sensitivity C-reactive protein (CRP) assay (cat. n. L2KCR2, Siemens, USA) was performed using the IMMULITE® 2000 Analyzer (DPC, Los Angeles, CA, USA), according to the manufacturer's protocol.

### Statistical Analysis

Statistical analyses were performed using the R statistical platform (https://www.R-project.org/) and the GraphPad 7.0 software (GraphPad Software Inc., La Jolla, Ca). D'Agostino-Pearson normality test was used to evaluate whether the continuous data were normally distributed, and according to the results, a Welch's two-tailed *t*-test for independent samples (for normally distributed data) or a Mann–Whitney *U*-test (for non-normally distributed data) was used. Multiple comparisons among more than two groups were made using the ANOVA test with Tukey's correction or the Kruskal Wallis test. The non-parametric Jonckheere-Terpstra test was used to analyze the trend between an ordinal independent variable. Categorical values were described by the number of occurrences and percentages and were compared using the chi-square test. Outliers have been detected and removed according to the ROUT method with *Q* coefficient 1%. To assess a correlation between cytokine levels and risk factors, a canonical correlation analysis (CCA) was performed (23). To investigate the collinearity effects among risk factors and food groups, data were analyzed with multivariate linear regression analysis. Generated models were analyzed with an ANOVA test to evaluate the goodness of fit. Regression coefficients were reported as an estimate and 95% confidence interval. Cytokine correlation matrix was obtained with Pearson's correlation test. Box plots denote median and 25th to 75th percentiles (boxes) and Tukey whiskers. *p*-value of <0.05 was considered statistically significant.

## Results

### Anthropometric and Clinical Characteristics of the Study Population

The enrolled population was represented by 150 blood donors, with 63 female participants and 87 male participants. Overall, the mean age was 40.9 years, while the BMI median was 25.92; 43 (28.66%) individuals were smokers (Table 1). Female and male populations did not display statistically significant differences for age, smoker percentage, physical activity (Table 1), cytometric blood counts, and biochemical analyses (Supplementary Table S2). However, the male population showed statistically significant higher weight, height, and BMI compared with the female population (Table 1).

**Table 1:** Clinical phenotyping of the population enrolled (*N* = 150).

**Parameters [unit]**	**Total population** **(*N* = 150)**	**Female population** **(*N* = 63; 42%)**	**Male population** **(*N* = 87; 58%)**	***p*-value**
**Age [years]**	40.9 ± 11.2	40.33 ± 11.84	39.62 ± 10.88	0.707^a^
**Weight [kg]**	75 [50; 120]	65 [50; 94]	82 [64; 120]	<0.0001 ^a^
**Height [m]**	170 [145; 186]	165 [145; 177]	175 [160; 186]	<0.0001 ^a^
**BMI [kg/m** ^**2**^ **]**	25.92 [19.16; 37.87]	23.44 [19.16; 36.51]	26.42 [20.98; 37.87]	0.0004 ^a^
**Smoker; yes (%)**	43 (28.66%)	22 (34.92%)	21 (24.14%)	0.2 ^b^
**Physical activity [MET]**	1,026 [0; 12,000]	1,026 [0; 11,784]	1,026 [0; 12,000]	0.828^a^

*Age is expressed as mean ± SD. Smokers are indicated as number and percentage. Other data are expressed as median and range [min; max]. MET, Metabolic EquivalenT, an indicative unit of physical activity (refer to the “MATERIALS AND METHODS” section)*.

*^a^Welch's test*.

*^b^Chi-square test*.

**Figure 1:**
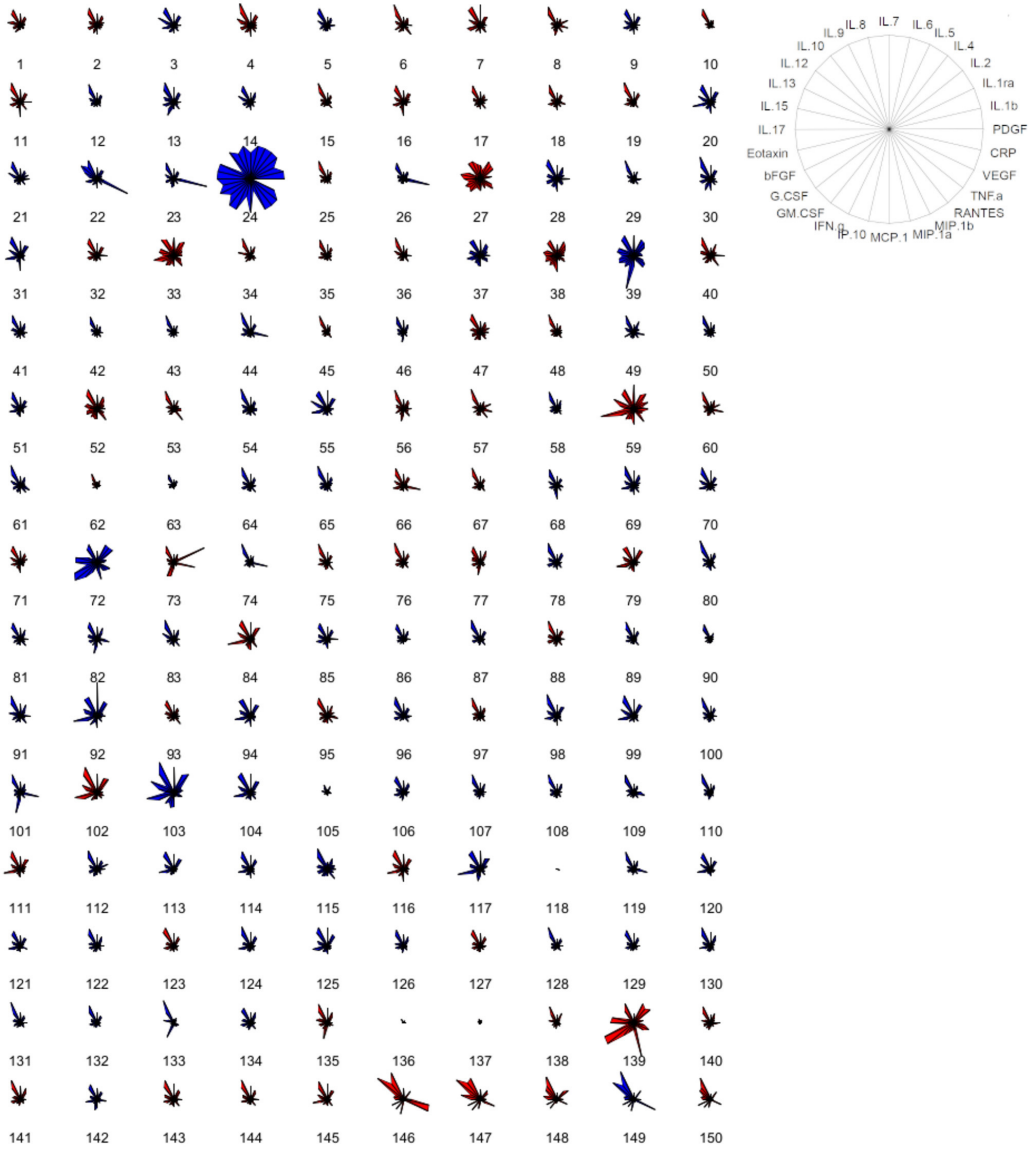
Cytokine-based pattern of volunteers. Star plot obtained by multivariate data analysis of the whole cytokinome of every subject, consisting of a sequence of equi-angular spokes (radii), with each spoke representing one cytokine, as indicated in the figure on the right. Data length of a spoke is proportional to the magnitude of the variable for the data point relative to the maximum magnitude of the variable across all data points. A line is drawn connecting the data values for each spoke. Blue stars represent male subjects, and red stars represent female subjects.

Serum samples of blood donors were then screened for the concentration of a panel of cytokines, chemokines, and growth factors and of C-reactive protein (hsCRP). Overall, all factors were detectable in serum specimens, with a defined cytokine/chemokine profile for every individual (Figure 1; Table 2); 22% of total individuals had hsCRP concentrations >3 mg/L. However, these subjects did not display any significant difference in cytokine concentration, compared to those with hsCRP <3 mg/L (Supplementary Table S3). No statistically significant differences between the male and female participants were observed for all circulating factors (Figure 1; Table 2).

**Table 2:** Serum concentration of cytokines, chemokines, and growth factors.

**Cytokine**	**Concentration** **(total)**	**Concentration** **(female)**	**Concentration** **(male)**	***p*-value**
**IL-1b**	1.89 [1.81; 2.22]	1.89 [1.81; 2.22]	1.89 [1.81; 2.22]	0.74
**IL-1ra**	352.5 [288.1; 463.6]	381.8 [284.4; 546.7]	341.7 [289.3; 400]	0.16
**IL-2**	15.46 [14.84; 17.20]	16.07 [14.52; 17.58]	15.46 [14.84; 16.68]	0.97
**IL-4**	5.93 [5.21; 7.22]	6.1 [5.27; 7.4]	5.81 [5.21; 7.04]	0.39
**IL-5**	55.51 [50.69; 61.5]	55.51 [49.42; 61.5]	55.51 [50.69; 61.5]	0.17
**IL-6**	7.85 [7.18; 8.91]	7.85 [7.29; 9.17]	7.85 [7.01; 8.91]	0.09
**IL-7**	43.69 [40.38; 46.93]	43.69 [40.38; 46.93]	43.69 [40.79; 48.52]	0.88
**IL-8**	18.82 [16.34; 23.02]	19.86 [16.19; 25.51]	18.56 [16.34; 20.98]	0.17
**IL-9**	252.1 [233.6; 264.4]	252.4 [234.6; 263.7]	251.3 [232.6; 266.2]	0.87
**IL-10**	24.85 [22.65; 26.9]	24.85 [22.65; 27.56]	24.85 [22.65; 26.73]	0.48
**IL-12**	11.26 [10.84; 12.88]	12.08 [11.26; 14.05]	11.26 [10.41; 12.08]	0.27
**IL-13**	5.41 [4.72; 6.08]	5.41 [4.72; 6.08]	5.41 [4.81; 6.16]	0.78
**IL-15**	354.9 [338.4; 379.4]	360.1 [344.1; 390.6]	349.6 [338.4; 372.4]	0.34
**IL-17**	26.28 [23.78; 30.3]	26.28 [23.56; 31.67]	26.69 [23.78; 29.51]	0.92
**EOTAXIN**	47.79 [33.82; 63.48]	47.62 [29.51; 59.58]	47.88 [36.96; 67.44]	0.4
**b-FGF**	78.43 [73.34; 85.86]	78.43 [73.34; 87.07]	78.43 [73.34; 83.41]	0.25
**G-CSF**	293.3 [260.3; 353.7]	283.8 [249; 360.1]	293.3 [263.1; 352.4]	0.75
**GM-CSF**	12.89 [12.45; 13.47]	12.97 [12.45; 13.96]	12.75 [12.3; 13.18]	0.3
**IFN-γ**	12.44 [11.43; 13.47]	12.78 [11.56; 14.36]	12.24 [11.36; 13.84]	0.13
**IP-10**	250.5 [203.5; 306.5]	260.8 [202.8; 333.6]	242.5 [203.8; 281.1]	0.09
**MCP-1**	33.63 [26.58; 44.53]	33.74 [25.94; 43.64]	33.32 [27.65; 44.77]	0.72
**MIP-1α**	2.6 [2.39; 2.91]	2.66 [2.39; 3]	2.53 [2.39; 2.82]	0.36
**MIP-1β**	81.52 [75.51; 90.01]	81.72 [74.91; 87.94]	81.37 [76.52; 91.43]	0.5
**PDGF**	1,538 [1,180; 1,994]	1,497 [1,180; 2,114]	1,553 [1,151; 1,956]	0.92
**RANTES/ CCL5**	6,573 [4,951; 7,858]	6,843 [4,825; 8,706]	6,251 [5,150; 7,557]	0.32
**TNF-α**	48.87 [44.63; 55.39]	48.49 [44.63; 56.91]	49.26 [45.4; 55.39]	0.27
**VEGF**	416.9 [398.7; 446.9]	421.4 [402.2; 455]	412.5 [397.5; 436.5]	0.29
**CRP**	1.22 [0.57; 2.59]	1.1 [0.47; 2.61]	1.29 [0.76; 2.47]	0.99

*Cytokine, chemokine, and growth factor concentrations are expressed as pg/ml. hsCRP values are expressed as mg/L. Results are indicated as median and IQR [25th percentile; 75th percentile]*.

### Cytokines and LGCI Risk Factors

**Figure 2:**
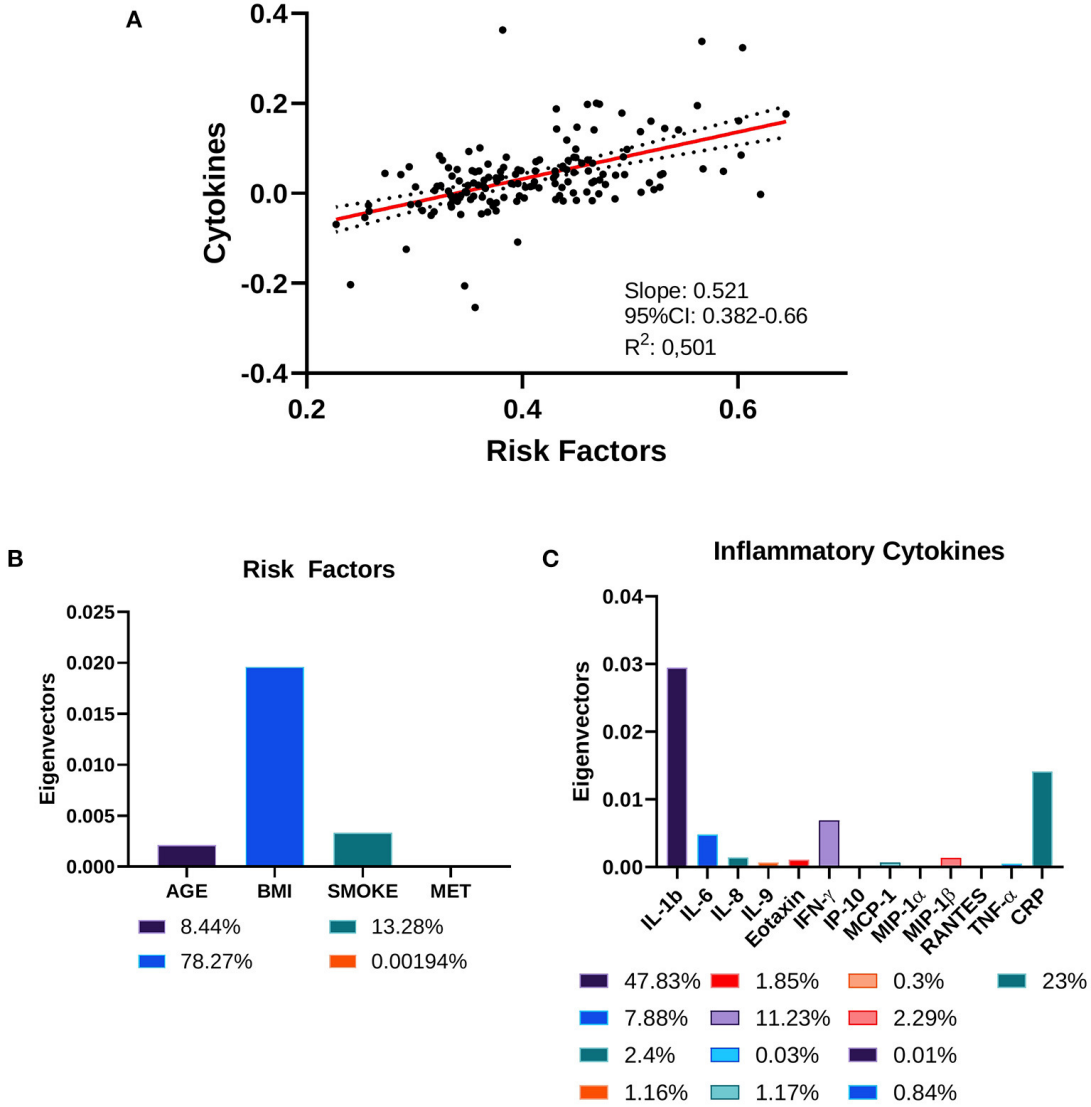
Canonical correlation analysis of cytokines and risk factors. **(A)** Two-way CCA setting; each subject is described by two canonical variates per mode, represented on a scatter plot. The two variate groups are maximally correlated, and their linear regression slope corresponds to the canonical correlation coefficient. **(B)** Risk factors' standardized weights and **(C)** cytokines' standardized weights are represented as absolute values and as percentage in the color legends.

Next, we investigated the correlation between LGCI risk factors (age, BMI, smoke, physical activity) and the inflammatory cytokines IL-1b, IL-6, IL-8, IL-9, eotaxin, IFN-γ, IP-10, MCP-1, MIP-1α, MIP-1β, RANTES, TNF-α, and CRP. The CCA revealed that LGCI risk factors and inflammatory cytokines displayed a correlation coefficient of 0.521 (95% CI: 0.382–0.66), with a goodness of fit equal to 0.501 (Figure 2A). The standardized weights for the single risk factors indicated a stronger relevance of BMI, which concurred for 78.27% of the correlation; smoke contributed to 13.28%, while age and physical activity contributed to 8.44% and <0.002%, respectively (Figure 2B). The standardized weights for the single inflammatory cytokines showed that IL-1b, CRP, IFN-γ, and IL-6 were the principal components of the correlation, concurring for 47.83, 23, 11.23, and 7.88%, respectively (Figure 2C).

**Figure 3:**
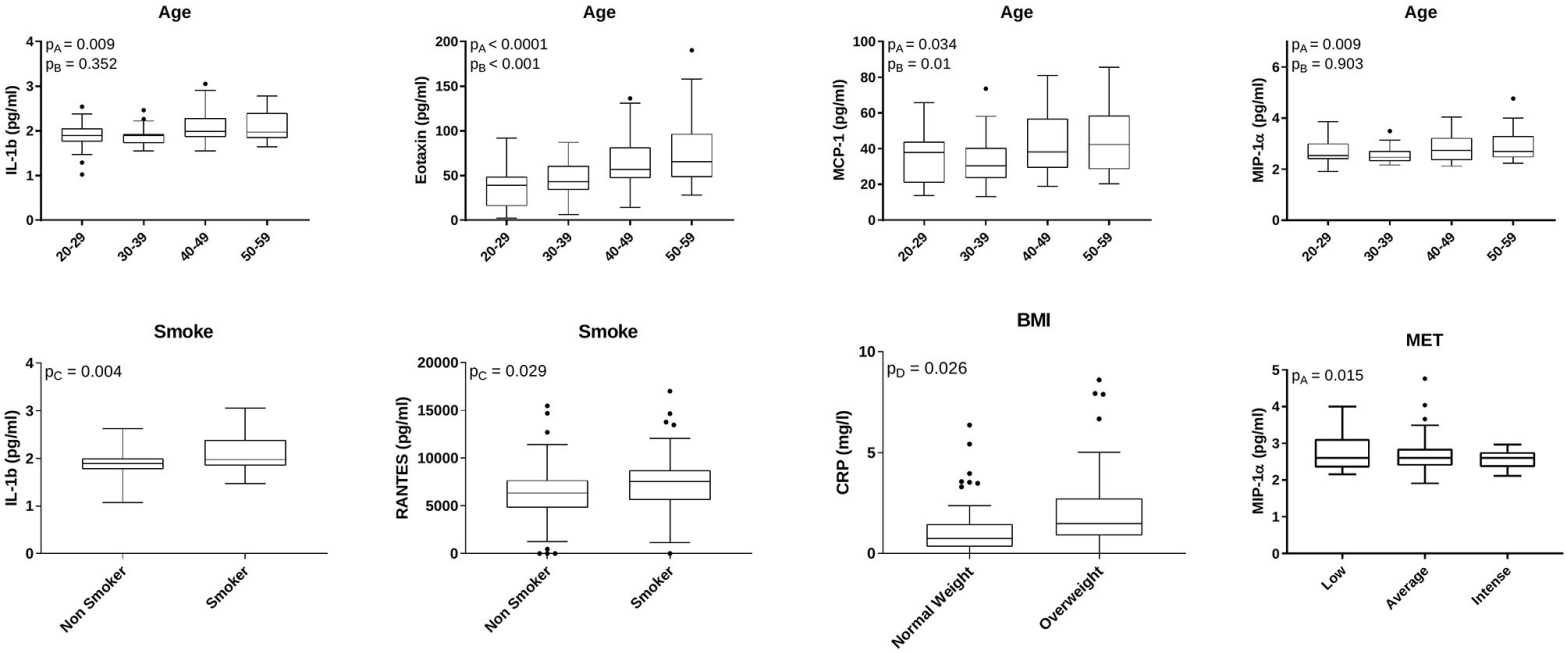
Age, smoke, BMI, and physical activity-related cytokines. Box plots indicate cytokine concentrations in subjects classified for age (*N* = 29: 20–29 years; *N* = 53: 30–39 years; *N* = 33: 40–49 years; *N* = 29: 50–59 years), smoke habit (*N* = 107 non-smokers; *N* = 43 smokers), BMI (*N* = 65 normal weight; *N* = 60 overweight), and physical activity (*N* = 74 low physical activity; *N* = 53 average physical activity; *N* = 23 intense physical activity). IL-1b, eotaxin, MCP-1, MIP-1α, and CCL5/RANTES concentrations are expressed as pg/ml. CRP concentration is expressed as mg/L. The figure reports only factors with statistically significant different values. *p*A = overall ANOVA test; *p*B = Jonckheere-Terpstra trend test; *p*C = Welch's *t*-test for paired distributions; *p*D = Mann–Whitney test for non-parametric distributions.

Next, the association between specific cytokines and LGCI-risk factors was investigated. Interestingly, IL-1b, eotaxin, MCP-1, and MIP-1α displayed a significant overall difference among four age-related groups (i.e., 20–29, 30–39, 40–49, and 50–59 years). Eotaxin and MCP-1 showed also an increasing trend associated with age (20–29 ≤ 30–39 ≤ 40–49 ≤ 50–59 years; Figure 3). In smokers, a significant increase in IL-1b and RANTES/CCL5 was detected (Figure 3). Overweight individuals (BMI: 25–30) showed significantly higher levels of CRP, compared with normal weight individuals (BMI: 20–24.9). Finally, physical activity was associated with higher levels of MIP-1α (Figure 3).

### Cytokines and Diet

**Figure 4:**
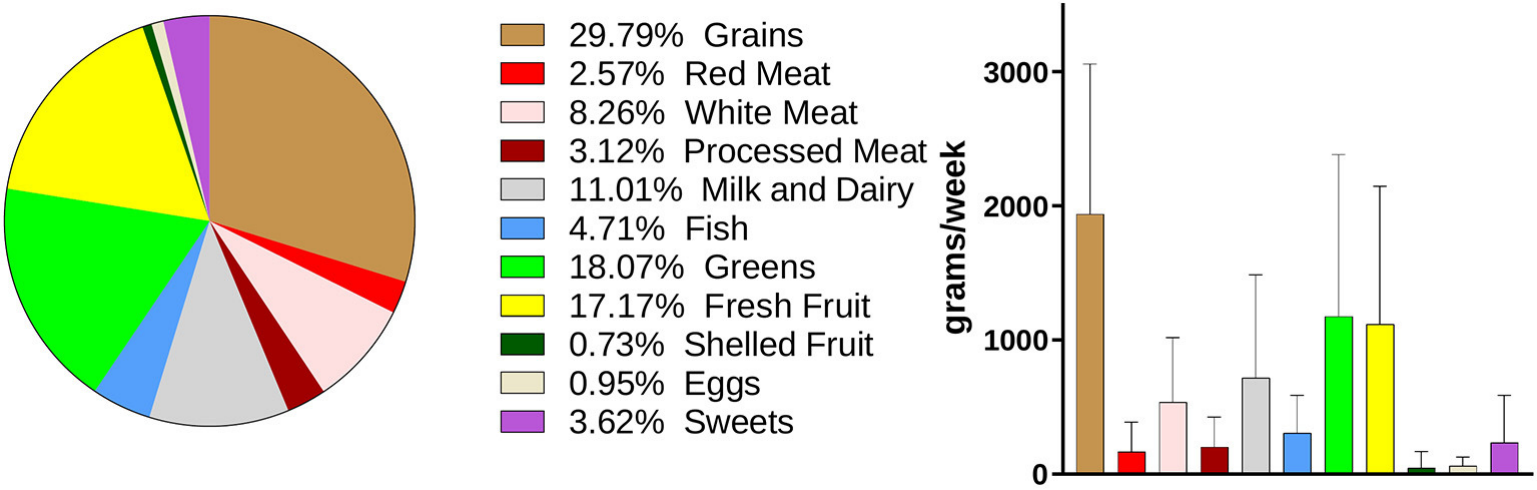
Dietary habits of the enrolled volunteers. Weekly consumption of foods is expressed in grams. Data are showed in a pie-chart as percentage compared with the whole weekly food intake and in a histogram as mean ± SD for the single food groups.

Dietary information was collected with a weekly food-frequency questionnaire, as described in the “Materials and Methods” section and in Supplementary Table S1. Data relative to the weekly total consumption of each food item (in g) were collected, included in 11 food groups, and analyzed. The main components of the dietary intake of the volunteers were “Grains,” “Greens,” “Fresh Fruits,” and “Milk and Dairy,” which accounted for 29.79, 18.07, 17.17, and 11.01% of the total food consumption, respectively (Figure 4).

**Figure 5:**
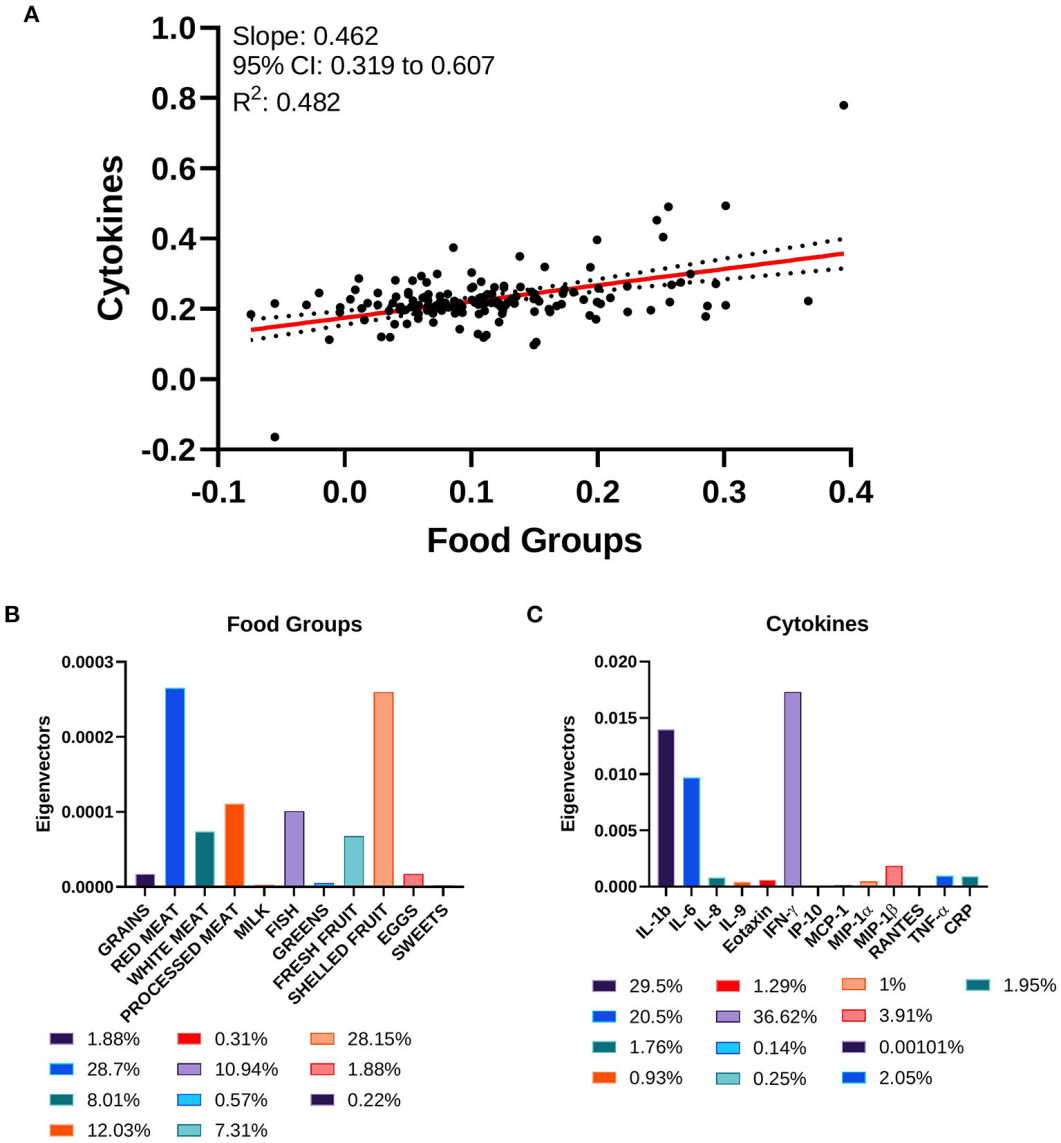
Canonical correlation analysis of cytokines and food groups. **(A)** Two-way CCA setting; each subject is described by two canonical variates per mode, represented on a scatter plot. The two variate groups are maximally correlated, and their linear regression slope corresponds to the canonical correlation coefficient. **(B)** Food groups' standardized weights and **(C)** cytokines' standardized weights are represented as absolute values and as percentage in the color legends.

Food groups and inflammatory cytokines displayed a correlation coefficient of 0.462 (95% CI: 0.319–0.607), with a goodness of fit equal to 0.482 (Figure 5A). The standardized weights for the single food items indicated a stronger relevance of Red Meats and Shelled Fruits, which concurred for the 28.7 and 28.15% of the correlation, respectively (Figure 5B). Among the inflammatory cytokines, IFN-γ, IL-1b, and IL-6 were the principal components of the correlation, contributing to 36.62, 29.5, and 20.5%, respectively (Figure 5C).

**Figure 6:**
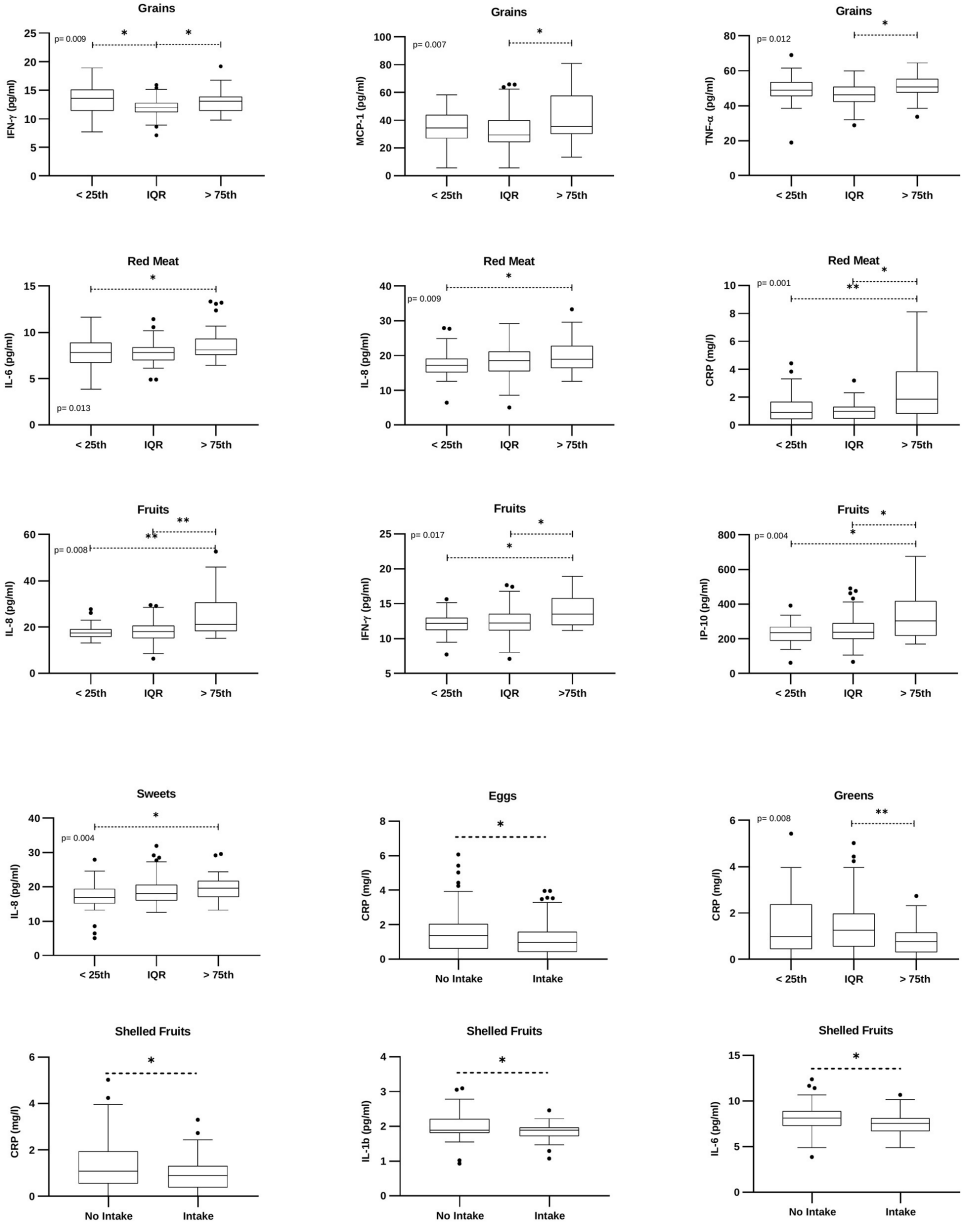
Food groups-related cytokines. Box plots denote cytokine concentrations in subjects with low (“ <25th” – subjects with a weekly intake lower than the 25th percentile), medium (“IQR” – subjects with a weekly intake between the 25th and the 75th percentile), or higher (“>75th” – subjects with a weekly intake higher than the 75th percentile) food intake; or intake/no intake of a specific food group. Cytokine concentrations are expressed as pg/ml. CRP concentration is expressed as mg/L. The figure reports only factors with statistically significant values. *p* = Brown-Forsythe ANOVA or Kruskal Wallis test. *p <0.05; **p <0.01.

At univariate analysis, individuals with a weekly grain intake higher than the 75th percentile displayed significantly higher levels of circulating IFN-γ, MCP-1, and TNF-α (Figure 6). The intake of red meat was associated with a significant increase in IL-6, IL-8, and CRP (Figure 6). Moreover, subjects who ate more fruits showed higher levels of IL-8, IFN-γ, and IP-10 (Figure 6). Increased levels of IL-8 were detected also in subjects consuming more sweets (Figure 6). A significant reduction in CRP was observed in individuals who ate more eggs, greens, or shelled fruits. In these latter subjects, a significant reduction of IL-1b and IL-6 was also found (Figure 6).

### Multivariate Analysis and Cytokine Correlations in Healthy Subjects

In multivariate analysis, all significant associations among cytokines and lifestyle factors or food groups found at univariate analysis (Figures 3, 6) were still retained upon adjusting for BMI (Supplementary Table S4).

Among all cytokines, MCP-1, IL-1b, and CRP levels were significantly associated both with risk factors and with specific food groups (Figures 3, 6). As shown in Table 3, the association between MCP-1 and the intake of grains was not significant after adjusting for age; similarly, the association between IL-1b and shelled fruits was not significant when adjusted for age and smoke. Interestingly, CRP levels were not associated with the consumption of red meat, shelled fruits, eggs, and greens after adjusting for BMI (Table 3).

**Table 3:** Multivariate linear regression analyses between risk factors and food groups for MCP-1, IL-1b, and CRP.

**ANOVA model comparisons**				**Intercept**		**Risk factor**		**Food group**		**Interaction**	
**Model 1**	**Model 2**	***p*-Value**	**Best fitting** **model**	**Estimate** **(95% CI)**	***p*-Value**	**Estimate** **(95% CI)**	***p*-Value**	**Estimate** **(95% CI)**	***p*-Value**	**Estimate** **(95% CI)**	***p*-Value**
MCP-1 ~ age + grains	MCP-1 ~ age * grains	0.486	Model 1	16.76 (5.98 – 27.5)	0.0025	0.356 (0.130 −0.581)	0.0022	3.06 (−0.213 to 6.33)	0.07	–	–
IL-1b ~ age + smoke + shelled fruit	IL-1b ~ (age + smoke) * shelled fruit	0.158	Model 1	1.66 (1.48 −1.85)	<2^−16^	Age 0.007 (0.0023–0.012)	Age 0.0029	3.06 (−0.219 to 6.34)	0.07	–	–
						Smoke 0.141 (0.03–0.252)	Smoke 0.014			–	–
CRP ~ BMI + shelled fruit+ eggs + red meat + greens	CRP ~ BMI * (shelled fruit + eggs + red meat + greens)	0.301	Model 1	−0.662 (−2.24 to 0.92)	0.413	0.356 (0.132–0.58)	0.022	Shelled fruit −0.32 (−1.07 to 1.05)	Shelled fruit 0.082	–	–
								Eggs −0.054 (−1.67 to 0.432)	Eggs 0.77	–	–
								Red meat −0.12 (−0.263 to 2.65)	Red meat 0.574	–	–
								Greens −0.243 (−0.577 to 0.92)	Greens 0.061	–	–

*For each linear regression, two models have been evaluated and compared: Model 1 considering variables without interactions and Model 2 considering both variables and interactions. Models have been compared with an ANOVA test, whose output revealed if the differences among the two models were statistically significant (p-value <0.05). The best-fitting model has been chosen accordingly, Coefficients for intercept, risk factor(s), and food group(s) have been shown for the best-fitting model*.

**Figure 7:**
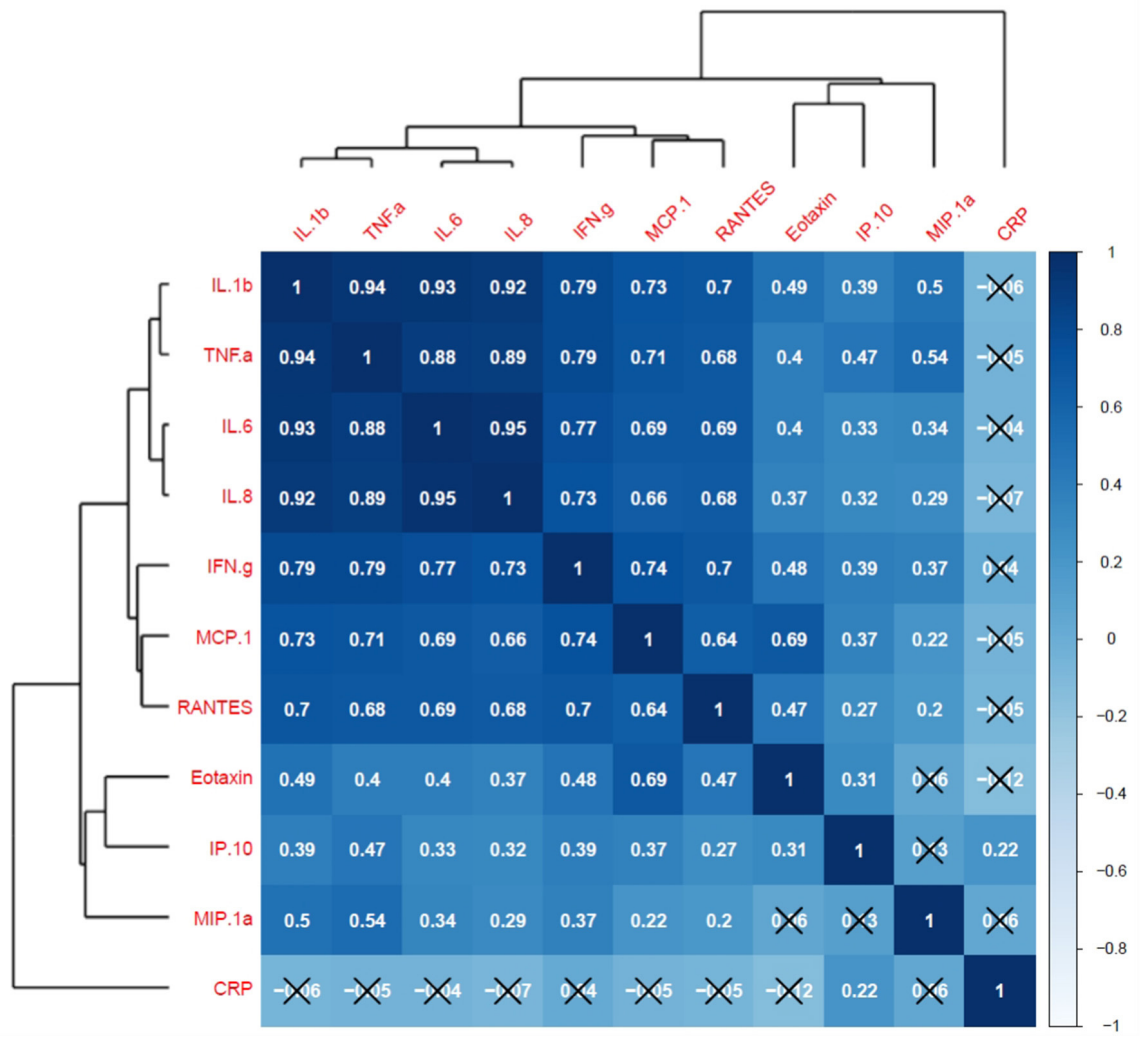
Cytokine correlation matrix and hierarchical clustering. Pearson's correlation coefficients for cytokine's scaled measurements are visualized by both tile-color intensity (according to the legend on the right) and by the *r*-values inside the tiles. *r*-values closer to 1 show a positive correlation; *r*-values closer to −1 show a negative correlation; *r*-values closer to 0 denote the absence of a correlation among the considered variables. All values not labeled with a black X are statistically significant (*p*-value <0.05). Dendrograms on the left and on top of the matrix show the hierarchical clustering.

Finally, a correlation matrix based on cytokines significantly associated with at least one LGCI-related risk factor (age, BMI, smoke, physical inactivity, diet) indicated positive and significant correlations among all selected inflammatory cytokines, except for CRP, for which almost no correlations with cytokines were observed (Figure 7). Interestingly, IL-1b, TNF-α, IL-6, and IL-8 established a cluster of cytokines with r-values ranging from 0.88 (CI: 0.84–0.91) to 0.95 (CI: 0.93–0.96), thus representing a potential LGCI-related cytokine pattern biomarker (Figure 7, Supplementary Table S5).

## Discussion

Cytokines are key mediators in inflammatory, viral, and autoimmune diseases, as well as in LGCI (4–6, 10, 17, 24). However, it is still difficult to use cytokines as diagnostic or prognostic tools due to the problem of establishing “normal” vs. “abnormal” cytokine levels. Few studies investigated cytokine concentrations in healthy subjects, and often a limited number of variables have been examined.

In this study, we have analyzed the cytokine profile of a cohort of 150 healthy blood donors. We have addressed how age, BMI, smoke, physical activity, and dietary habits impact on specific cytokines, modulating their concentrations. We have shown that each individual has a peculiar cytokine pattern, influenced at a different degree, by physiologic and behavioral factors. No differences between male and female participants were detected, despite the different BMI. These results are in agreement with other studies performed with different populations (25, 26) and suggest that other factors, such as hormones, may balance the control elicited by BMI.

The BMI has the heaviest impact on the correlation between LGCI-related risk factors and cytokines. Indeed, many studies have characterized the cytokine profile in subjects with obesity and/or severe obesity detecting differences compared with normal weight individuals (10, 27, 28). In this study, we have found that, despite the removal from the analysis of subjects with obesity, BMI was still significantly associated with CRP levels. Interestingly, we have observed that CRP levels correlated also with the consumption of specific foods, i.e., eggs, red meat, shelled fruits, and greens; however, when corrected for BMI, all these associations were lost, thus highlighting the impact of the body mass.

The second most involved factor in the control of cytokine profile is tobacco use. Smokers displayed higher levels of IL-1b and CCL5/RANTES. These findings are in line with *in vivo* studies showing that cigarette smoke induces murine emphysema with lung inflammation and DNA injury/apoptosis *via* IL-1b and CCL5-CCR5 (29, 30). However, CCL5 exerts also regenerative functions, mainly through CCR1 (31). Thus, its increase may also suggest the activation of tissue repair mechanisms.

Age contributes 8.4% to the correlation between LGCI risk factors and inflammatory cytokines. Inflamm-aging is a dysregulation of the cytokine network well described at cellular levels, where cell senescence is associated with a defined secretory phenotype (SASP) characterized by specific cytokines, such as IL-6, IL-8, and VEGF (32). At serum levels, data are sometimes conflicting since some studies have enrolled different aged people, without considering health status. Other studies have been performed on very old people, such as non-agenarians and centenarians (33). In this study, we have shown that 20–60 years old healthy subjects display an age-associated increase of IL-1b, eotaxin, MCP-1, and MIP-1α. Accordingly, eotaxin levels have been found to increase in 21–86 and in 7–17 years old subjects, compared with 1–6 years old children (26).

Finally, the contribution of physical activity has been evaluated. A general idea coming from recent literature data indicates that exercise stimulates both pro- and anti-inflammatory cytokines (IL-6, IL-8, IL-10, TNF-α), whose levels return to baseline sometime from 5 to 24 h after exercise (13). The dual response depends also on the type of exercise (aerobic or anaerobic) and whether it comes from short-term or long-term physical activity (34). In this study, we have observed that people who in the 7 days before the questionnaire consumed more *Metabolic EquivalenT (MET)*, a unit used to express energy spent and oxygen burned, had higher levels of MIP-1α, a protein mainly produced by macrophages and involved in leucocyte recruitment (35). Its increase may in part explain the mobilization of different leukocyte populations to the blood observed in diverse studies focused on the effect of exercise on blood cells and molecules (13).

Healthy diets are commonly considered protective for LGCI. Anti-inflammatory properties have been conferred to single foods (i.e., almonds, yogurt, nuts, dark chocolate, and extra virgin olive oil), functional foods (i.e., omega-3 fatty acids, polyphenols, and fibers), and whole diets, such as Mediterranean diet (1, 7, 36–38). To quantify the dietary quality, various dietary indices or scores have also been reported. For instance, the Dietary Inflammatory Index (DII®) is a literature-derived method based on 45 food parameters (39), the Healthy Eating Index-2015 (HEI-2015) is a measure of dietary quality according to the Dietary Guidelines for Americans (DGA) (15), the polyphenol antioxidant content (PAC) score is an index of the total content of diet in polyphenols (16), and the empirical dietary inflammatory pattern (EDIP) is a score based on the reduced rank regression approach, developed in the United States and validated across nations (40). Moreover, some indices have been conceived around specific dietary patterns, such as the Mediterranean diet and the Dietary Approaches to Stop Hypertension (DASH) (14, 41).

The main result of our work is the finding that 7-day consumption of particular food items is associated with increased levels of specific cytokines. All volunteers were from Southern Italy, a place where the food culture adheres to the Mediterranean diet. Indeed, the main components of the dietary intake of the volunteers were grains followed by greens, fresh fruit, milk and dairy, and white meat. The beneficial effect of this diet is represented by the result that in this population, no associations were detected between specific food consumption and CRP levels (when adjusted for BMI). At present, there are few studies and some discrepancies about the intake of grains. Some studies reported an anti-inflammatory effect of whole grains for their nutrients and fibers; other studies, performed with healthy subjects, displayed no effects (42, 43). Moreover, the daily consumption of wheat products and cereals has been reported to contribute to chronic inflammation and autoimmune diseases (42). Our data indicate that volunteers with grain consumption over the 75th percentile had higher levels of TNF-α and IFN-γ, compared with those in the IQR range, representing 50% of the population. TNF-α increase may be associated with carbohydrate-related hyperglycemia, which has been shown to increase circulating cytokine concentrations by an oxidative mechanism (44). IFN-γ was increased also in subjects with grain consumption under the 25th percentile. This low inflammatory profile may be due to the large use of cereals and of whole-wheat pasta and bread. Red meat and shelled fruit, even though represented a small percentage of the whole dietary intake, showed the greatest influence on the correlation between foods and inflammatory cytokines. Subjects with red meat consumption over the 75th percentile displayed the most striking inflammatory pattern, characterized by the increase in IL-6 and IL-8. These data are in agreement with other reports showing that a higher intake of meat, as in Western-like diets, is associated with inflammation and detrimental health outcome (7).

Fruits and vegetables, rich in flavonoids and antioxidants, have been associated with a lower risk of LGCI-related diseases and low levels of some markers of inflammation (45, 46). Surprisingly, volunteers who had a large consumption of fresh fruits displayed higher circulating levels of IL-8, IFN-γ, and IP-10, compared to those with a moderate or low intake. Different studies suggest an anti-inflammatory role for polyphenols, elicited mainly through the modulation of NF-kB and MAPK pathways at multiple levels (46). However, clinical trials are somewhat contradictory, and *in vitro* studies have also reported an induction of IL-8 expression by resveratrol, a polyphenol contained in many fruits, thus opening new questions (46, 47). Moreover, IL-8 and IP-10 release may be induced by IFN-γ, whose increase could suggest an immuno-stimulatory effect of the fruit. Interestingly, the intake of shelled fruits was associated with a significant decrease of the most pro-inflammatory molecules IL-1b and IL-6. These results are in line with previous studies showing an anti-inflammatory and antioxidant role for almonds and nuts (48, 49).

Thus, in a healthy population, age, BMI, smoke, physical activity, and certain dietary habits are associated with specific cytokines. These associations remained also upon the adjustment for BMI. However, CRP, the classical marker of acute inflammation, was associated only with the BMI and did not correlate with the other cytokines. Thus, although CRP is a valuable indicator of inflammation, other molecules may be useful as markers, particularly to identify the relative weight of specific factors involved in the progression of LGCI. This is of particular importance when considering dietary habits. In this study, we have identified IL-1b and IL-6 as the cytokines with the strongest relevance in the correlation between the cytokinome and LGCI risk factors. These molecules are considered inflammatory markers for multiple conditions (17, 50) and, together with TNF-α and IL-8, establish a cluster that may represent a potential LGCI-related cytokine biomarker.

In conclusion, within this study, we have provided evidence for the measurement of multiple cytokines in a well-defined and characterized healthy population and how lifestyle factors and aging affect specific cytokines.

Future research directions will expand the dietary analysis by considering the percentage of macronutrients, mainly the type of fats (i.e., omega 3 and omega 6) that have displayed a relevant impact on circulating cytokine concentrations (51, 52). Moreover, the impact of lifestyle/dietary factors in the framework of international studies on healthy and pathological populations will be addressed. Finally, intervention studies will further define the contributions of the examined factors on cytokine levels. Results will allow to envision new technological tools and digital platforms to early detect mediators and risk factors of LGCI and to encourage healthy lifestyle behaviors.

## Data Availability Statement

The raw data supporting the conclusions of this article will be made available by the authors, without undue reservation.

## Ethics Statement

The studies involving human participants were reviewed and approved by Ethical Committee of the University of Naples Federico II. The patients/participants provided their written informed consent to participate in this study.

## Author Contributions

Conceptualization: PF and MLi. Software: MS and FC. Formal analysis: MDT and MS. Investigation: MLe, SC, GP, BC, and SM. Data curation: MDT, VD'E, MRA, and AP. Writing—original draft preparation: VD'E, MDT, and MLe. Writing—review and editing: VD'E, PF, MLi, and FC. Supervision: PF and MS. Project administration and funding acquisition: PF and VD'E. All authors have read and agreed to the published version of the manuscript.

## Funding

This research was funded in part by Regione Campania POR FESR 2014-2020-Objective 1.2 – Realization of Technology Platform to fight oncologic diseases (RARE PLAT NET, SATIN, and COEPICA projects) and by the Italian Association for the Cancer Research – AIRC (grant IG19001).

## Conflict of Interest

FC was employed by the Anti-inflammaging Company AG. The remaining authors declare that the research was conducted in the absence of any commercial or financial relationships that could be construed as a potential conflict of interest.

The reviewer GA declared a shared affiliation with the authors MDT, MLe, SC, AP, BC, GP, MS, and PF to the handling editor at the time of review.

## Publisher's Note

All claims expressed in this article are solely those of the authors and do not necessarily represent those of their affiliated organizations, or those of the publisher, the editors and the reviewers. Any product that may be evaluated in this article, or claim that may be made by its manufacturer, is not guaranteed or endorsed by the publisher.
