# Supplementary material for: Lifestyle and Dietary Habits Affect Plasma Levels of Specific Cytokines in Healthy Subjects
Source: Front Nutr. 2022 Jun 24;9:913176. doi: 10.3389/fnut.2022.913176 (PMC9270017; doi:10.3389/fnut.2022.913176)
Supplement: Supplementary file 1 [file Data_Sheet_1.docx]

| **Supplementary Table 1.** Food Items and classification. | | | |
| --- | --- | --- | --- |
| **Food Items** | **No. of pictures** | **Additional Questions** | **Food Category** |
| Cornflakes | 3 | YES | Grains |
| Pasta | 3 | YES | Grains |
| Rice | 3 | YES | Grains |
| Potatoes | 3 | YES | Grains |
| Barley | 3 | NO | Grains |
| Spelt | 3 | NO | Grains |
| Crackers | 3 | YES | Grains |
| Bread | 3 | YES | Grains |
| Pizza | 2 | YES | Grains |
| Chicken | 3 | YES | White Meat |
| Turkey | 3 | YES | White Meat |
| Lamb | 3 | YES | White Meat |
| Rabbit | 3 | YES | White Meat |
| Pork | 3 | YES | White Meat |
| Beef | 3 | YES | Red Meat |
| Cured meats and salami | 3 | YES | Processed Meat |
| Fish | 3 | YES | Fish |
| Canned Fish | 2 | YES | Fish |
| Crustaceans | 3 | YES | Fish |
| Cheese | 1 | YES | Milk and Dairy |
| Seasoned Cheese | 3 | YES | Milk and Dairy |
| Eggs | 1 | YES | Eggs |
| Legumes (pulses) | 3 | NO | Grains |
| Lentils | 3 | NO | Grains |
| Beans | 3 | NO | Grains |
| Chickpeas | 3 | NO | Grains |
| Soy | 3 | NO | Grains |
| Almonds | 3 | NO | Shelled Fruit |
| Nuts | 3 | NO | Shelled Fruit |
| Walnuts | 3 | NO | Shelled Fruit |
| Pine nuts | 3 | NO | Shelled Fruit |
| Pistachios | 3 | NO | Shelled Fruit |
| Fruit vegetables | 3 | YES | Fresh Fruit |
| Raw vegetables | 3 | YES | Greens |
| Milk | 3 | YES | Milk and Dairy |
| Yogurt | 3 | YES | Milk and Dairy |
| Pastries/Ice creams | 3 | YES | Sweets |
| Sweet biscuits | 3 | NO | Sweets |
| Cakes | 3 | NO | Sweets |

| **Supplementary Table 2.** Blood count and biochemical analysis of the enrolled population**.** Data are expressed as median and range [min; max]. | | | |
| --- | --- | --- | --- |
| **Complete Blood Count** | **Range Values (Total)** | **Range Values (Female)** | **Range Values (Male)** |
| **RBC (10^6^/µl)** | 5.05 [3.91; 7.25] | 4.51 [3.91; 5.67] | 5.23 [4.39; 7.25] |
| **Hb (gr/dl)** | 14.7 [12; 20.3] | 13.35 [12; 16.6] | 15.2 [13.1; 20.3] |
| **HCT (%)** | 43.8 [35.6; 60.1] | 40.3 [35.6; 49.5] | 44.4 [39.5; 60.1] |
| **MCV (fl)** | 86.9 [69.4; 95.2] | 88.85 [79.9; 95.2] | 85.4 [69.4; 94.2] |
| **MCH (pg)** | 29.5 [21.5; 31.8] | 29.7 [25.7; 31.1] | 29.2 [21.5; 31.8] |
| **MCHC (g/dl)** | 33.6 [31; 35.8] | 33.1 [31.8; 35.7] | 33.8 [31; 35.8] |
| **PLT (10^3^/µl)** | 232 [135; 364] | 227.5 [135; 364] | 234 [135; 313] |
| **WBC (10^3^/µl)** | 6.2 [3.33; 12.5] | 6.435 [3.97; 11.4] | 6.15 [3.33; 12.5] |
| **Neutrophils (%)** | 57.1 [30.5; 73.9] | 58.2 [3.05; 73.9] | 55.8 [32.7; 68.8] |
| **Lymphocytes (%)** | 31.9 [26.6; 52.6] | 32.55 [2.66; 49.6] | 31.9 [22.7; 52.6] |
| **Monocytes (%)** | 7.4 [0.48; 11.9] | 6.75 [0.48; 8.8] | 7.8 [4.7; 11.9] |
| **Eosinophils (%)** | 2.4 [0.1; 12] | 1.6 [0.1; 6.1] | 2.6 [0.6; 12] |
| **Basophils (%)** | 0.6 [0.03; 1.4] | 0.55 [0.03; 1.4] | 0.6 [0.2; 1.2] |
| **Neutrophils/Lymphocytes ratio** | 1.79 [0.62; 3.62] | 1.77 [0.75; 3.62] | 1.79 [0.62; 3.03] |
| **AST (UI/l)** | 23 [15; 41] | 21 [16;29] | 23 [15; 41] |
| **ALT (UI/l)** | 19 [8; 52] | 15 [8; 30] | 20 [10; 52] |
| **Cholesterol (mg/dl)** | 177 [115; 268] | 190 [132; 268] | 174 [115; 253] |
| **Triglycerides (mg/dl)** | 75 [42; 234] | 61 [42; 188] | 79 [43; 234] |
| **Serum Iron (μg/dl)** | 95 [43; 271] | 97 [46; 271] | 93.5 [43; 228] |
| **Total Proteins (g/dl)** | 7.1 [6.4; 7.9] | 7.2 [6.8; 7.7] | 7.1 [6.4; 7.9] |
| **Creatinine (mg/dl)** | 0.88 [0.52; 1.12] | 0.64 [0.52; 0.96] | 0.905 [0.58; 1.12] |

| Supplementary Table 3. Serum concentration of cytokines in subjects with LOW CRP (< 3 mg/l, n = 117) and HIGH CRP (>3 mg/l, n = 33). Cytokine, chemokine and growth factor concentrations are expressed as pg/ml. Results are indicated as median and IQR [25th percentile; 75th percentile]. | | | |
| --- | --- | --- | --- |
| Cytokine | **Low CRP** | **High CRP** | **p-value** |
| IL-1b | 1.89 [1.81; 2.22] | 1.89 [1.81; 2.10] | 0.747 |
| IL-1ra | 333 [265; 450] | 400 [340; 564] | 0.065 |
| IL-2 | 16.1 [14.7; 17.3] | 15.5 [14.5; 16.2] | 0.315 |
| IL-4 | 6.1 [5.35; 7.4] | 5.62 [4.69; 6.53] | 0.072 |
| IL-5 | 55.5 [50.7; 61.9] | 53.9 [49.4; 56.7] | 0.125 |
| IL-6 | 7.85 [7.08; 8.91] | 8.12 [7.15; 9.04] | 0.418 |
| IL-7 | 43.7 [41.6; 48.5] | 42 [40.4; 45.3] | 0.098 |
| IL-8 | 19.1 [16.3; 24.2] | 17.5 [15.5; 21.7] | 0.268 |
| IL-9 | 252 [233; 266] | 247 [235; 262] | 0.746 |
| IL-10 | 25.5 [22.8; 27.9] | 24.1 [22.7; 26.2] | 0.113 |
| IL-12 | 11.3 [11.3; 12.9] | 11.3 [10.4; 13.3] | 0.376 |
| IL-13 | 5.41 [4.72; 6.08] | 5.07 [4.72; 5.92] | 0.260 |
| IL-15 | 355 [343; 383] | 344 [333; 365] | 0.082 |
| IL-17 | 27.1 [23.8; 31.9] | 24.6 [23.1; 27.9] | 0.143 |
| Eotaxin | 49.7 [35.2; 65.2] | 40.6 [28.8; 56] | 0.065 |
| b-FGF | 78.4 [73.3; 85.9] | 78.4 [72; 83.4] | 0.673 |
| G-CSF | 293 [258; 368] | 291 [262; 337] | 0.622 |
| GM-CSF | 12.9 [12.5; 13.6] | 12.6 [12.5; 13.3] | 0.405 |
| IFN-γ | 12.2 [11.2; 13.8] | 12.9 [12.1; 14.6] | 0.106 |
| IP-10 | 243 [198; 305] | 261 [211; 365] | 0.144 |
| MCP-1 | 32.6 [26.4; 45.1] | 37.6 [26.4; 43.6] | 0.509 |
| MIP-1α | 2.53 [2.37; 2.93] | 2.66 [2.53; 2.85] | 0.178 |
| MIP-1β | 81.4 [75.3; 91.3] | 82.7 [76.2; 87.3] | 0.289 |
| RANTES | 6251 [4890; 7846] | 6754 [5427; 8110] | 0.458 |
| PDGF | 1553 [1156; 1986] | 1485 [1127; 2076] | 0.736 |
| TNF-α | 47.7 [44.6; 55.4] | 49.3 [45.4; 55.4] | 0.515 |
| VEGF | 419 [399; 446] | 417 [399; 452] | 0.950 |

| **Supplementary Table 4**. Multivariate linear regression analyses between Cytokines and Risk Factors or Food Groups, adjusted for BMI. For each linear regression, two models have been evaluated and compared: Model 1 refers to a linear regression model among the specified Parameter and Cytokines; Model 2 refers to the same linear regression model adjusted for BMI. For both models, β coefficient with 95% Confidence Intervals are shown. Models have been compared with an ANOVA whose output revealed if the differences among the two models were statistically significant (p-value < 0.05). | | | | |
| --- | --- | --- | --- | --- |
| **Parameter** | **Cytokine** | **β Model 1 [95% CI]** | **β Model 2 [95% CI]** | **ANOVA p-val** |
| Age | IL-1b | 0.00394  [-0.0182 to 0.0260] | 0.00356 [-0.0189 to 0.0260] | 0.826 ns |
|  | Eotaxin | 0.875 [0.447 to 1.30] | 0.921 [0.490 to 1.35] | 0.157 ns |
|  | MCP-1 | 0.276  [-0.00305 to 0.555] | 0.280 [-0.00373 to 0.563] | 0.872 ns |
|  | MIP-1α | -0.884 [-2.74 to 0.973] | -0.919 [-2.80 to 0.965] | 0.805 ns |
| Smoke | IL-1b | 0.596 [0.0558 to 1.14] | 0.597 [0.0547 to 1.14] | 0.772 ns |
|  | RANTES | 2080 [741 to 3420] | 2082 [738 to 3425] | 0.750  ns |
| MET | MIP-1α | -0.00594 [-0.0157 to 0.0037] | -0.00593 [-0.0157 to 0.0038] | 0.955  ns |
| Grains | IFN-γ | 0.000322 [-0.000240 to 0.000884] | 0.000279 [-0.000287 to 0.000845] | 0.246 ns |
|  | MCP-1 | 0.00337 [0.000571 to 0.00616] | 0.00340 [0.000571 to 0.000623] | 0.862 ns |
|  | TNF-α | 0.00189 [-0.00452 to 0.0083] | 0.00187 [-0.00461 to 0.00836] | 0.967 ns |
| Red Meat | IL-6 | 0.00205 [-0.00356 to 0.00767] | 0.00191 [-0.00372 to 0.00754] | 0.420 ns |
|  | IL-8 | 0.0154 [-0.0721 to 0.103] | 0.0155 [-0.724 to 0.103] | 0.948 ns |
| Fresh Fruits | IL-8 | 0.00877 [-0.00988 to 0.0274] | 0.00877 [-0.00995 to 0.0275] | 0.981 ns |
|  | IFN-γ | 0.000751 [0.000150 to 0.00135] | 0.000760 [0.000161 to 0.00136] | 0.170 ns |
|  | IP-10 | 0.0371 [0.0155 to 0.0588] | 0.0373 [0.0155 to 0.0590] | 0.595 ns |
| Sweets | IL-8 | -0.0127 [-0.0676 to 0.0421] | -0.0130 [-0.0685 to 0.0424] | 0.922 ns |
| Shelled  Fruits | IL-1b | 0.121 [-0.417 to 0.659] | 0.137 [-0.41 to 0.685] | 0.726  ns |
|  | IL-6 | 1.33 [-1.33 to 4] | 1.57 [-1.13 to 4.28] | 0.301  ns |

| **Supplementary Table 5.** Cytokine Correlation Matrix. Pearson’s correlation coefficients for cytokine’s scaled measurements by r-values and 95% Confidence Intervals. r-values closer to 1 show a positive correlation; r-values close to -1 show a negative correlation; r-values close to 0 denotes the absence of a correlation among the considered variables. | | | | | | | | | | | |
| --- | --- | --- | --- | --- | --- | --- | --- | --- | --- | --- | --- |
|  | **Il-1b** | **TNF-α** | **IL-6** | **IL-8** | **IFN-γ** | **MCP-1** | **RANTES** | **Eotaxin** | **IP-10** | **MIP-1α** | **CRP** |
| **IL-1b** | 1 | 0.94  [0.92; 0.96] | 0.93 [0.91; 0.95] | 0.92 [0.89; 0.94] | 0.79 [0.73; 0.85] | 0.73 [0.64; 0.79] | 0.7 [0.61; 0.78] | 0.49 [0.36; 0.6] | 0.39 [0.24; 0.51] | 0.5 [0.37; 0.61] | -0.06 [-0.22; 0.10] |
| **TNF-α** | 0.94  [0.92; 0.96] | 1 | 0.88 [0.84; 0.91] | 0.89 [0.85; 0.92] | 0.79 [0.73; 0.85] | 0.71 [0.62: 0.78] | 0.68 [0.59;  0.76] | 0.4 [0.26;  0.53] | 0.47 [0.33; 0.58] | 0.54 [0.41; 0.64] | -0.05 [-0.21; 0.11] |
| **IL-6** | 0.93 [0.91; 0.95] | 0.88 [0.84; 0.91] | 1 | 0.95 [0.93; 0.96] | 0.77 [0.7; 0.83] | 0.69 [0.59;  0.76] | 0.69 [0.59; 0.76] | 0.4 [0.25;  0.52] | 0.33 [0.18;  0.46] | 0.34 [0.19; 0.48] | -0.04 [-0.2; 0.12] |
| **IL-8** | 0.92 [0.89; 0.94] | 0.89 [0.85; 0.92] | 0.95 [0.93; 0.96] | 1 | 0.73 [0.65; 0.8] | 0.66 [0.56; 0.74] | 0.68 [0.58; 0.76] | 0.37 [0.22; 0.5] | 0.32 [0.17; 0.46] | 0.29 [0.14; 0.43] | -0.07 [-0.22; 0.1] |
| **IFN-γ** | 0.79 [0.73; 0.85] | 0.79 [0.73; 0.85] | 0.77 [0.7; 0.83] | 0.73 [0.65; 0.8] | 1 | 0.74  [0.66; 0.81] | 0.7 [0.6; 0.77] | 0.48 [0.34; 0.59] | 0.39 [0.25;  0.52] | 0.37 [0.22; 0.5] | 0.04 [-0.13; 0.19] |
| **MCP-1** | 0.73 [0.64; 0.79] | 0.71 [0.62: 0.78] | 0.69 [0.59;  0.76] | 0.66 [0.56; 0.74] | 0.74  [0.66; 0.81] | 1 | 0.64 [0.54; 0.73] | 0.69 [0.59; 0.76] | 0.37 [0.22; 0.5] | 0.22 [0.06; 0.36] | -0.05 [-0.2; 0.12] |
| **RANTES** | 0.7 [0.61; 0.78] | 0.68 [0.59;  0.76] | 0.69 [0.59; 0.76] | 0.68 [0.58; 0.76] | 0.7 [0.6; 0.77] | 0.64 [0.54; 0.73] | 1 | 0.47 [0.33; 0.58] | 0.27 [0.11; 0.41] | 0.2 [0.04; 0.35] | -0.05 [-0.21; 0.11] |
| **Eotaxin** | 0.49 [0.36; 0.6] | 0.4 [0.26;  0.53] | 0.4 [0.25;  0.52] | 0.37 [0.22; 0.5] | 0.48 [0.34; 0.59] | 0.69 [0.59; 0.76] | 0.47 [0.33; 0.58] | 1 | 0.31 [0.15; 0.45] | 0.06 [-0.1; 0.22] | -0.12 [-0.28; 0.04] |
| **IP-10** | 0.39 [0.24; 0.51] | 0.47 [0.33; 0.58] | 0.33 [0.18;  0.46] | 0.32 [0.17; 0.46] | 0.39 [0.25;  0.52] | 0.37 [0.22; 0.5] | 0.27 [0.11; 0.41] | 0.31 [0.15; 0.45] | 1 | 0.13 [-0.04; 0.28] | 0.22  [0.06; 0.36] |
| **MIP-1α** | 0.5 [0.37; 0.61] | 0.54 [0.41; 0.64] | 0.34 [0.19; 0.48] | 0.29 [0.14; 0.43] | 0.37 [0.22; 0.5] | 0.22 [0.06; 0.36] | 0.2 [0.04; 0.35] | 0.06 [-0.1; 0.22] | 0.13 [-0.04; 0.28] | 1 | 0.06 [-0.22; 0.1] |
| **CRP** | -0.06 [-0.22; 0.10] | -0.05 [-0.21; 0.11] | -0.04 [-0.2; 0.12] | -0.07 [-0.22; 0.1] | 0.04 [-0.13; 0.19] | -0.05 [-0.2; 0.12] | -0.05 [-0.21; 0.11] | -0.12 [-0.28; 0.04] | 0.22  [0.06; 0.36] | 0.06 [-0.22; 0.1] | 1 |
